# Controlling the optical properties of nanostructured oxide-based polymer films

**DOI:** 10.1038/s41598-021-94881-3

**Published:** 2021-08-06

**Authors:** N. C. Angastiniotis, S. Christopoulos, K. C. Petallidou, A. M. Efstathiou, A. Othonos, L. Koutsokeras

**Affiliations:** 1 Department of Mechanical Engineering and Materials Science and Engineering, Cyprus University of Technology, Limassol, Cyprus; 2 Department of Sciences and Engineering, Sorbonne University Abu Dhabi, Abu Dhabi, United Arab Emirates; 3 Department of Chemistry, Heterogeneous Catalysis Laboratory, University of Cyprus, Nicosia, Cyprus; 4 Department of Physics, University of Cyprus, Nicosia, Cyprus

**Keywords:** Materials for devices, Materials for optics, Nanoscale materials, Structural materials

## Abstract

A bulk scale process is implemented for the production of nanostructured film composites comprising unary or multi-component metal oxide nanoparticles dispersed in a suitable polymer matrix. The as-received nanoparticles, namely Al$_2$O$_3$, SiO$_2$ and TiO$_2$ and binary combinations, are treated following specific chemical and mechanical processes in order to be suspended at the optimal size and composition. Subsequently, a polymer extrusion technique is employed for the fabrication of each film, while the molten polymer is mixed with the treated metal oxide nanoparticles. Transmission and reflection measurements are performed in order to map the optical properties of the fabricated, nanostructured films in the UV, VIS and IR. The results substantiate the capability of the overall methodology to regulate the optical properties of the films depending on the type of nanoparticle formation which can be adjusted both in size and composition.

## Introduction

Continuous development of advanced technologies in recent years has yielded the combined use of finely-divided inorganic nanoparticle fillers in polymer resins^1–4^. Such materials are known to exhibit unique, via regulation, optical, mechanical and thermal properties among others and, as a result, are widely applied in the automotive, aerospace, electronics, military, clothing and agricultural sectors. Regulation of the physical properties arises from our ability to manipulate the characteristics of the utilized nanoparticle and polymer species, in particular the concentration, the size, the shape, the interfacial characteristics and the degree of dispersion of nanoparticles in the polymer matrix. Nonetheless, one of the greatest challenges in achieving a homogeneous dispersion of uniformly sized nanoparticles in a polymer matrix is to circumvent the notorious tendency of nanoparticles to form clusters and agglomerates^5,6^. Even though, for instance, such materials are characterized by enhanced infrared absorption, their use has been proved problematic, mainly due to increased scattering (i.e. haze)^7–10^, caused by inorganic particles. Furthermore, the array of inorganic particles is often poor, suffering from inhomogeneity, limited compositions, as well as sizes and unwanted agglomeration^11–13^, which leads to non-uniform properties.

The work hereby presented overcomes the current limitations through the use of a multistage, controllable process, to homogeneously disperse, in a suitable polymer film, inorganic nanoparticle formations of unary or diverse composition that can deliver predefined functionality. As each particle formation can be forced to assume certain characteristics in virtue of their composition and type of treatment, the properties of the film can be regulated accordingly. Characterization of the optical properties of the as-fabricated, nanostructured films substantiate the capability of the overall methodology verifying that the optical properties of the films can be regulated depending on the type of nanoparticle formation, which can be adjusted preferentially both in size and composition. As a proof of principle, the present study focuses on TiO$_2$, Al$_2$O$_3$ and SiO$_2$ nanoparticle–polymer composites, due to the wide and well-established use of the active media^14–16^. It is expected that the nanocomposite films presented here will be widely utilized in various sectors of everyday life and industry such as agriculture, construction, screen/TV technology, photography, etc.

## Sample preparation and experimental techniques

### Material composition

In the work presented herein three types of nanopowders are used: silica-nanopowder (Sigma-Aldrich, p.n. 718483) with 12 nm average particle size and 175–225 m$^2$/g surface area, titanium (IV) oxide anatase-nanopowder (Sigma-Aldrich, p.n. 637254) with $<25$ nm particle size and 45–55 m$^2$/g surface area and aluminum oxide (Alfa Aesar, p.n. 44931) with 40–50 nm particle size and surface area 32–45 m$^2$/g. Water based suspensions of the as-received powders are prepared by applying planetary and/or ball milling for deagglomeration while adding suitable amounts of a poly(acrylic acid) dispersant (PAA). The dispersant is required to keep the particles uniformly distributed, while certain compositions include different powders, thus enabling high particle concentration at low viscosity. The separation of particles in solution, allows the desired mixing of the comprising nanospecies, whether unary or heterogeneous. Equimolar binary suspensions of the aforementioned nanopowders are also prepared, thus bringing the total number of suspensions to six, i.e., three unary (Al$_2$O$_3$, SiO$_2$ and TiO$_2$) and three heterogeneous (Al$_2$O$_3$–SiO$_2$, Al$_2$O$_3$–TiO$_2$ and TiO$_2$–SiO$_2$). Once prepared, the suspensions are subsequently wet sieved, freeze granulated and freeze dried under vacuum, before undergoing extrusion. It is noted that, the fabricated suspensions are designed to pronounce potential synergy effects on the optical properties of the nanocomposite films.

**Table 1:** Powder mass (g) on a per suspension basis.

Powder (g)	Suspension type					
	Al$_2$O$_3$–TiO$_2$	Al$_2$O$_3$–SiO$_2$	TiO$_2$–SiO$_2$	Al$_2$O$_3$	TiO$_2$	SiO$_2$
Al$_2$O$_3$	84.11	94.38		150		
TiO$_2$	65.89		85.60		150	
SiO$_2$		55.62	64.40			150

### Dispersion

The PAA is a commercial product and is provided in solution with 25 wt% concentration. It is an ammonium water soluble salt of poly(acrylic acid), with ammonia (NH$_4^+$) as counter ion to the COO– groups. Being one of the most common types of dispersants for ceramic powders in water^17–19^, the PAA can readily be adsorbed on the particle surfaces thus allowing for electrosteric stabilization. The electrostatic repulsion is produced by the negatively charged COO– groups and the steric repulsion by the polymer chains in the water. Normally, the dissociation of the PAA-salt is complete at high pH values (9–10), whereas at lower values it is significantly reduced and therefore gives a poor stabilizing effect. The PAA with ammonia as counter ion increases the pH values up to 9–10^17,18^. Powders with very acidic properties, such as silica^17,18^, produce lower pH values, which is overcome by incrementing gradually, on an empirical basis, the amount of powder in a finite amount of PAA. By following the above procedure, the predetermined amount of each powder type, as indicated in Table 1, is dispersed in its respective suspension. The PAA and powder concentrations for each suspension type are presented in Table 2. In general, for a powder with a specific surface area of 10 m$^2$/g, the typical amount of PAA is 0.3 wt%, i.e. 0.3 mg/m$^2$. Adsorption, however, can vary much from one material to the other. Optimizing the amount and type of dispersant entails an extensive amount of work, as for each type/amount, a separate suspension needs to be prepared and evaluated, preferably by rheological and viscosity measurements. In the framework of this work though, there was no need for optimizing the nanopowder dispersion, albeit the latter could be enhanced. Using the here tabulated quantity of PAA, the nanoparticles remained dispersed in water for several hours, before initiating the granulation. The suspensions were 45 $\upmu$m—wet sieved prior to granulation to safe-guard the nozzle.

**Table 2:** PAA and powder concentrations on a per suspension basis.

	Suspension type					
	Al$_2$O$_3$–TiO$_2$	Al$_2$O$_3$–SiO$_2$	TiO$_2$–SiO$_2$	Al$_2$O$_3$	TiO$_2$	SiO$_2$
PAA (g)	10	14.4	13.6	10	10	10
Solid PAA (wt%)	1.64	2.34	2.22	1.64	1.64	1.64
H$_2$O (g)	153.4	507.2	541.2	189	193	1010
Powder (wt%)	47.86	22.33	21.28	42.98	42.49	12.72
Powder (vol%)	18.91	7.62	7.46	15.92	16.00	5.21

Note: PAA (g) refers to the solution of PAA. Solid PAA (wt%) is calculated with respect to the total mass of solid components in solution. The vol% and wt% refer to the powder in solution.

### Milling

The milling was used as type of mechanical treatment for deagglomeration of the as-received powders and not for reducing the primary particle size. In the case of Al$_2$O$_3$ and TiO$_2$, the planetary milling was performed at 200 rpm in a container, where 2/3 of the powder was initially milled for 30 min. The rest of the powder was added subsequently, and the milling was allowed to continue for an additional interval of 90 min. For the Al$_2$O$_3$–TiO$_2$ mixed oxide, the planetary milling was performed at 200 rpm in a container, where Al$_2$O$_3$ was initially milled for 30 min, while TiO$_2$ was subsequently added, and the milling resumed for 90 min. For the SiO$_2$, after the initial planetary milling, the solution had to be additionally ball milled due to the severe need for dilution. The powder was added gradually in regulated portions for a 40 h milling. Finally, in the case of the mixed oxides, Al$_2$O$_3$–SiO$_2$ and TiO$_2$–SiO$_2$, after Al$_2$O$_3$ and TiO$_2$ were planetary milled separately, they were ball milled with the addition of SiO$_2$, while diluting with water and adding PAA.

**Table 3:** The atomization parameters on a per type of granule basis.

Granulation	Suspension type					
	Al$_2$O$_3$–TiO$_2$	Al$_2$O$_3$–SiO$_2$	TiO$_2$–SiO$_2$	Al$_2$O$_3$	TiO$_2$	SiO$_2$
Feed (l/h)	40	80	80	40	40	40
Air pressure (bar)	0.3	0.4	0.4	0.3	0.3	0.3

### Freeze granulation

As mentioned above, after the milling and prior to granulation, suspensions, wet seived at 45 $\upmu$m, were prepared. The slurries were then sprayed into liquid nitrogen forming frozen granules, with size in the range of 20–300 $\upmu$m. Due to the freezing process being spontaneous, degraded heat was produced, thus enhancing even further the homogeneity of the frozen granules. The average granule size was controlled by the atomization parameters, i.e., the suspension feed (pump speed) and the atomization air pressure, as presented in Table 3.

### Freeze drying

**Figure 1:**
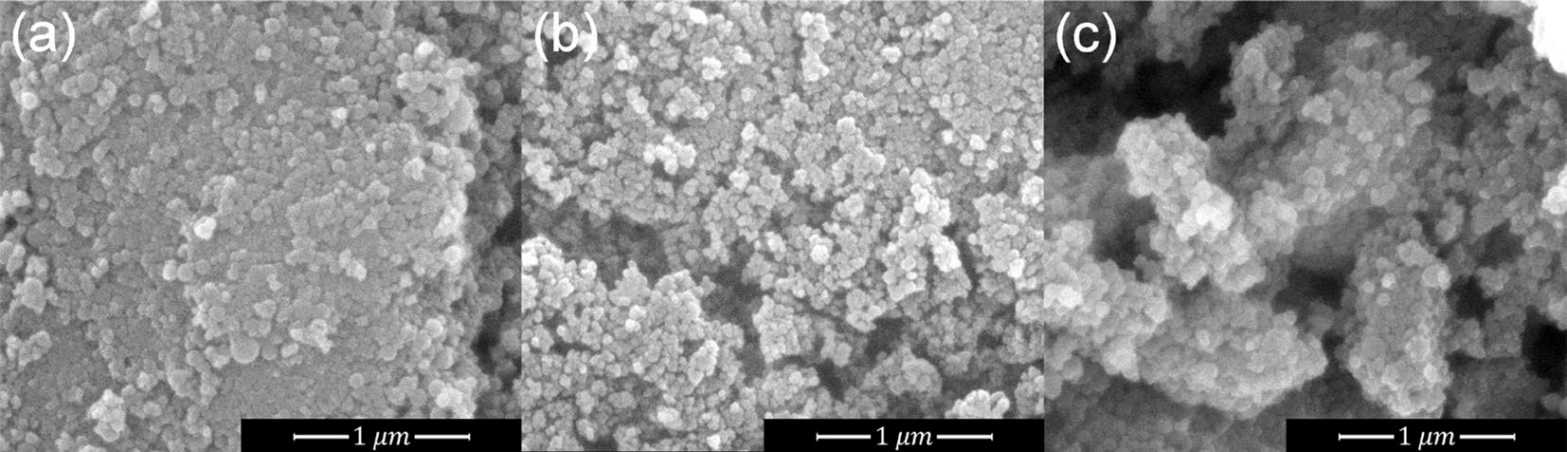
SEM micrographs of the freeze dried heterogenous granules: (**a**) Al$_2$O$_3$–SiO$_2$, (**b**) TiO$_2$–SiO$_2$ and (**c**) Al$_2$O$_3$–TiO$_2$.

Following the granulation process, the frozen granules were transferred to a freeze dryer, where they were instantaneously exposed to $-16\;{^\circ }$C at 1.5 mbar. During this highly irreversible process the water is rapidly removed by sublimation. This ensures that no migration phenomenon occurs, the homogeneity is enhanced due to production of entropy, and no hard binding within the granules is created. Scanning electron microscopy (SEM) was used for the morphological characterization of the freeze dried granules. The samples were sputter-coated, using a SC7640 Sputter coater, with a thin film of Au, to prevent charging, and then loaded to a FEI Quanta 200 microscope, mounted on aluminum specimen stubs using carbon stickers. Finally, images were acquired at 25 kV accelerating voltage and at various magnifications. Representative SEM photomicrographs of the freeze dried granules are shown in Fig. 1. The freeze-drying process, apart from preserving the homogeneity, allows weak interparticle bonding, which is here a deliberately sought after characteristic, as the granules were to be subsequently redispersed and disintegrated via extrusion into the low density polyethylene (LDPE), down to their as-suspended size. Energy Dispersive X-rays (EDX) technique, along with the SEM system, has been employed to investigate the elemental composition of the granulated powders. The results, not presented here for brevity, indeed verify the relative concentration of each nanoparticle species in the heterogeneous granules.

### Blending

**Figure 2:**
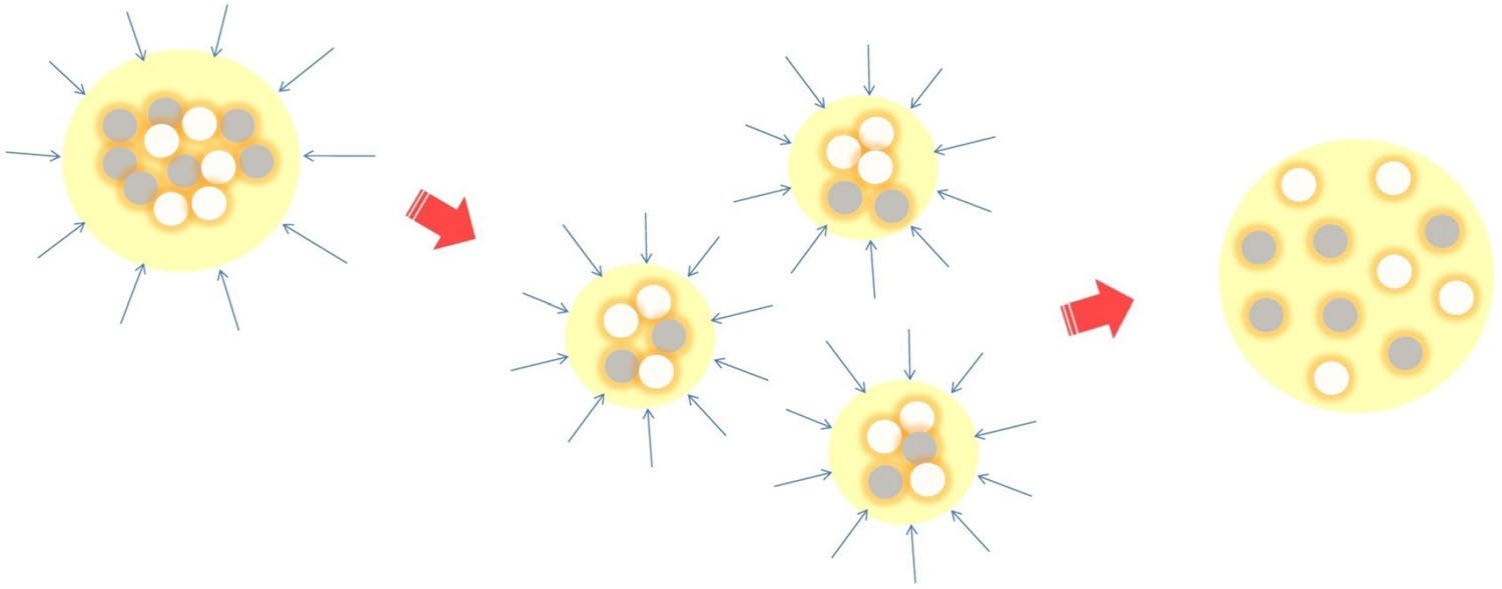
A heterogeneous granule is progressively divided into smaller fragments while being under uniform pressure (blue arrows) during the extrusion process. The large yellow colored spheres represent the linear low density polyethylene, whereas the grey and white colored circles represent two types of nanoparticles. The orange color coating encompassing the nanoparticles is the thin PAA film, present during the extrusion process as long as the temperature is kept below 250 ${^\circ }$C.

The low density polyethylene is a widely used, low cost polymer^20^, whose extrudability and optical properties, discussed below, make it an ideal candidate to act as a host for the granulated powders. However, the LDPE and the poly(acrylic acid) are totally immiscible due to their different molecular structures^21^ and their weak adhesion, due to the latter being polar, while the former is not^22^. We proceeded with the extrusion process at a polyethylene temperature of 250 ${^\circ }$C, ensuring the stability of the PAA film encompassing the nanoparticles during dispersion^23,24^, and thus facilitating the production of a homogeneous nanocomposite film. While conforming to the temperature limitation, six master batches were prepared as follows: (a) three master batches with 20 wt% active ingredient in LDPE, each one containing a unique granule of a single oxide (TiO$_2$, Al$_2$O$_3$ or SiO$_2$), and (b) three master batches with 20 wt% active ingredient in LDPE, each one containing a unique granule with an equimolar binary combination of TiO$_2$–Al$_2$O$_3$, TiO$_2$–SiO$_2$ and Al$_2$O$_3$–SiO$_2$. The disintegration of heterogeneous granules during the extrusion process is schematically represented in Fig. 2. As shown, a granule is progressively divided into smaller fragments until the individual nanoparticles of different composition are finally released in the polymeric matrix. The master batch percentage used in the fabrication of prototype films was 10%, thus reducing the amount of the active ingredient to 2 wt%. The prototype films produced have a thickness of 70 $\upmu$m. In addition, one batch was prepared using pure LDPE, in order to establish a reference.

## Results and discussion

The XRD patterns of the films and powders were acquired in a theta–theta diffractometer (Rigaku Ultima IV, Japan) equipped with a Cu tube, operated at 40 kV and 40 mA, using a parallel X-ray beam (Cu $K_{\alpha }$, $\lambda = 0.1542$ nm) conditioned by an X-ray mirror. The patterns were collected in conventional Bragg–Brentano scans in the range of $10{^\circ }$–$50{^\circ }$ $2\theta$, with $0.05{^\circ }$ step and speed of $0.3{^\circ }$ $2\theta$/min.

**Figure 3:**
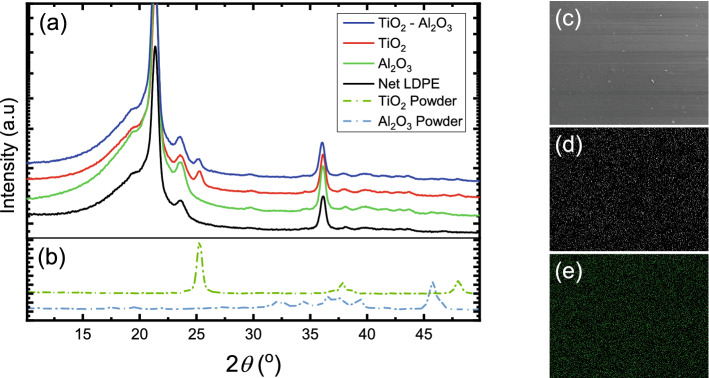
(**a**) XRD patterns of Net LDPE and of TiO$_2$, Al$_2$O$_3$ and TiO$_2$–Al$_2$O$_3$ nanoparticles embedded in LDPE from $10{^\circ }$ to $50{^\circ }$ $2\theta$. (**b**) XRD patterns of TiO$_2$ and Al$_2$O$_3$ granulated powders. (**c**) SEM image of an LDPE film containing TiO$_2$–Al$_2$O$_3$ nanoparticles and the corresponding EDX map showing the spatial distribution of the recorded X-ray intensity for (**d**) Ti and (**e**) Al. The area of the EDX map is approximately $1.6 \times 1.3$ mm$^2$.

Figure 3a,b presents the XRD patterns of selected samples. The granulated TiO$_2$ powder pattern, shown in Fig. 3b demonstrates the characteristic peaks of the metastable anatase phase with the (101) peak at $25.28{^\circ }$ $2\theta$ being the strongest in intensity. The FWHM of the (101) peak, as acquired and without any peak processing, has been found equal to $0.53{^\circ }$ $2\theta$, which is translated through the Scherrer equation^25^ to crystallite sizes of 16 nm. In Fig. 3b, the pattern of the granulated Al$_2$O$_3$ powder exhibits peaks assigned to the cubic $\gamma$-Al$_2$O$_3$ and orthorhombic $\delta$-Al$_2$O$_3$, indicating a mixture of the two phases, as specified by the supplier. However, due to multiple peak overlapping, the FWHM estimation and the crystallite size calculation for the Al$_2$O$_3$ powder is not feasible. The particle size though, is verified by the SEM images, shown in Fig. 1. Furthermore, the XRD pattern of the granulated SiO$_2$ powder, which is not shown here for brevity, demonstrates only a broad amorphous peak, centered at approximately $21{^\circ }$ $2\theta$, as expected. Pure LDPE is a semi-crystalline polymer, exhibiting an amorphous peak between $15{^\circ }$ and $25{^\circ }$ and four major peaks of the orthorhombic system, (110), (200), (210) and (020) at $21.4{^\circ }$, $23.6{^\circ }$, $29.8{^\circ }$ and $36.1{^\circ }$, respectively^26^, as shown in Fig. 3a. The XRD patterns of the TiO$_2$, Al$_2$O$_3$ and TiO$_2$–Al$_2$O$_3$ nanocomposite films are dominated by the characteristic peaks of the pure LDPE, while for the samples containing TiO$_2$ (unary and binary) only the major peak (101) is visible, at the same angular position. The FWHM of the (101) TiO$_2$ peak is $0.55{^\circ }$ and $0.45{^\circ }$ $2\theta$ for the films containing TiO$_2$ and TiO$_2$–Al$_2$O$_3$, respectively, indicating that the crystallite size remains unchanged during the extrusion process. On the other hand, the nanocomposite samples containing only Al$_2$O$_3$ do not exhibit any peak assigned to the nanoparticles due to the low intensity of the major peak of the granulated powder at $45.8{^\circ }$ $2\theta$.

Energy Dispersive X-rays (EDX) technique, along with the SEM system, has been also used to produce the elemental mapping of a film containing TiO$_2$–Al$_2$O$_3$ nanoparticles, at high and low magnifications. The results, presented in Fig. 3c–e, indeed verify a uniform and homogeneous dispersion of the nanoparticles in the polymer matrix.

UV–Vis transmission spectra were recorded in the 250–2500 nm range using a Perkin Elmer Lambda 950 spectrometer. An identical device was used, in diffuse reflectance setup, to record spectra of samples and reference material (fluorilon) from 250 to 800 nm (scan speed of 100 nm/min, slit width of 2.0 nm).

**Figure 4:**
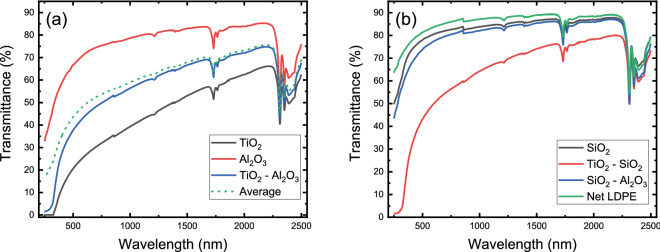
(**a**) Transmittance of TiO$_2$, Al$_2$O$_3$ and TiO$_2$–Al$_2$O$_3$ nanoparticles embedded in low density polyethylene (LDPE) from 250 to 2500 nm. The dashed line denotes the calculated average of the TiO$_2$ and Al$_2$O$_3$ curves. (**b**) Transmittance of SiO$_2$, TiO$_2$–SiO$_2$ and SiO$_2$–Al$_2$O$_3$ nanoparticles embedded in LDPE. The curve of net LDPE is given for comparison.

Experimentally determined transmittance spectra of the produced nanocomposite films are presented in Fig. 4a,b, on a cumulative basis. It is here noted that the results are reproducible for any chosen film spot. The Net LDPE spectrum exhibits a high transmittance plateau throughout the entire range, exhibiting only its characteristic absorption features at approximately 1730 nm, as well as after 2300 nm, visible in all nanocomposite films. Hence, it allows for direct observation of the spectral characteristics attributed to the addition of the granulated nanopowders in the polymer matrix. The TiO$_2$ profile substantiates the capability of the overall methodology as the UV wavelengths are totally absorbed, thus proving that the TiO$_2$ nanoparticles are uniformly dispersed in the entire body of the polyethylene matrix. Furthermore, the disintegration of the granulated particles consisting of equimolar proportions of TiO$_2$ and any of the other two oxides results again in a uniform distribution. This is evident through the total UV absorption which occurs independently of the reduced TiO$_2$ molar fraction due to the presence of the additional oxide. Even though the use of Al$_2$O$_3$ or SiO$_2$ in combination with TiO$_2$ does not affect the degree of UV absorption, the choice of Al$_2$O$_3$ or SiO$_2$ has a noticeable effect on the transmittance in the residual wavelength range. In Fig. 4a, the calculated average of the TiO$_2$ and Al$_2$O$_3$ transmittance curves follows the mixed oxide’s curve very closely, verifying the effect of Al$_2$O$_3$ in the composition. This is further established in Fig. 4b, where the use of SiO$_2$ and Al$_2$O$_3$ does not reduce the transmittance of net LDPE significantly. Therefore, a tailoring capability is implicit, as the molar ratios between the utilized species can be regulated a priori. Given the fact that the uniform dispersion in the polymer matrix can be ensured independently of the amount and type of the utilized species, the optical properties of the films in the visible and infrared wavelengths can be modified.

**Figure 5:**
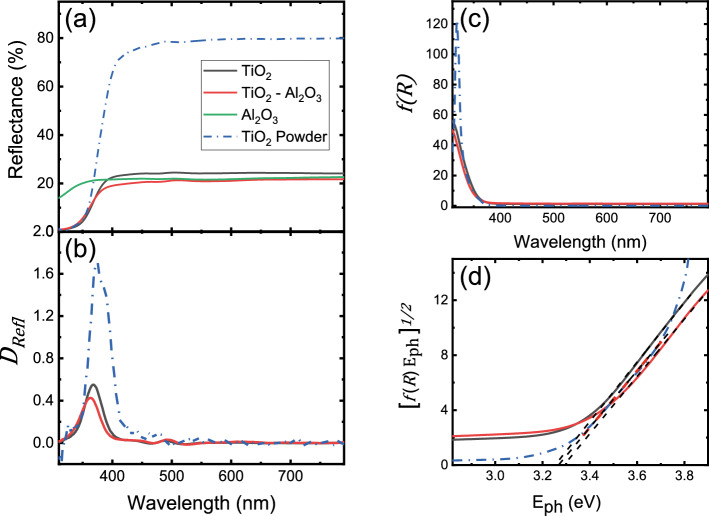
(**a**) Diffuse reflectance of TiO$_2$, TiO$_2$–Al$_2$O$_3$ and Al$_2$O$_3$ nanoparticles embedded in LDPE from 310 nm to 790 nm. The blue dashed-dot line denotes the diffuse reflectance of TiO$_2$ granulated powder. (**b**) The first derivative of the diffuse reflectance of TiO$_2$, TiO$_2$–Al$_2$O$_3$ films and TiO$_2$ granulated powder. (**c**) Kubelka–Munk function for each material. (**d**) Tauc plot of the reflectance spectrum with linear fits (black dashed lines) for each material. The horizontal axis intersection point of each linear fit denotes the corresponding energy band gap $E_g$.

Figure 5a presents the diffuse reflectance of TiO$_2$, TiO$_2$–Al$_2$O$_3$ and Al$_2$O$_3$ nanoparticles embedded in LDPE from 310 to 790 nm. A measurement on TiO$_2$ granulated powder is also provided for comparison. Measurements for wavelengths below 310 nm are not shown here due to low signal-to-noise ratio, originating from high absorption. It is evident that all materials containing TiO$_2$ follow very similar reflectance curves. Even though Al$_2$O$_3$ exhibits significantly higher reflectance in the UV, the absorption effects originating from the presence of TiO$_2$ dominate the spectral characteristics. In Fig. 5b, through the first derivative of the diffuse reflectance^27^, it becomes clear that in all TiO$_2$-containing materials, strong absorption effects occur for wavelengths around 400 nm. We subsequently calculate the pseudoabsorbance, represented by the Kubelka–Munk function $f(R_{\infty })$ according to the equation^28–30^:

1 $$\begin{aligned} f(R_{\infty })=\frac{(1-R_{\infty })^2}{2R_{\infty }}, \end{aligned}$$

where $R_{\infty }=\frac{R_{sample}}{R_{standard}}$ and $R_{sample}$, $R_{standard}$ are the reflectance of the sample and reference material, respectively. The observed behavior is thus further established by plotting the Kubelka–Munk function in Fig. 5c, which exhibits extremely similar characteristics for all three samples. We then apply the Tauc method, connecting the Kubelka–Munk function to the photon energy $E_{ph}$ and the optical band gap $E_g$ according to^30^:

2 $$\begin{aligned} \left( f(R_{\infty })\cdot E_{ph}\right) ^{1/n}=A\left( E_{ph}-E_g\right) , \end{aligned}$$

where *A* is an energy-independent constant and *n* is equal to 2 in the case of an indirect allowed transition, as expected for anatase TiO$_2$. Tauc plots of the reflectance spectra are shown in Fig. 5d, where an estimation of the energy band gap, $E_g$, of each material becomes feasible through the intersection of the horizontal energy axis with the corresponding linear fits^30,31^. Our calculations yield band gap energy values of $3.25\pm 0.01$ eV, $3.29\pm 0.01$ eV and $3.27\pm 0.01$ eV for TiO$_2$, TiO$_2$–Al$_2$O$_3$ and TiO$_2$ granulated powder, respectively. In accordance to other studies^32,33^, the extracted $E_g$ values strongly suggest that the primary particle size remains largely unaffected, while process-related changes occur only to the secondary particle size. Hence, the latter graphs prove that the process used to embed the nanoparticles in the polymer matrix does not lead to alteration of their spectral features. It is further noted here, that the use of SiO$_2$ instead of Al$_2$O$_3$ in the mixed oxide produces extremely comparable results. This is a major advantage of the method since it can be exploited in the a priori calculation of the relative fractions of the mixed oxides.

It is further noted that the scalability of the process has been already exemplified through the production of a large scale film, used as a greenhouse cover. The film has been tested and proven to exhibit significantly improved optical and thermal properties under environmental conditions, in comparison to conventional materials^34^. The active material chosen for the large scale film was the unary TiO$_2$ granule comprising nanoparticles of 25 nm.

**Table 4:** Key features of alternative fabrication techniques.

Technique	Key feature			
	Particle size control	Uniform dispersion	Combination of multiple species	Cost efficiency
Direct solvent blending	$\checkmark$	$\checkmark$	$\checkmark$	$\times$
Direct melt blending	$\checkmark$	$\times$	$\checkmark$	$\checkmark$
In-situ formation	$\checkmark$	$\checkmark$	$\times$	$\times$
Hybrid core–shell	$\checkmark$	$\checkmark$	$\times$	$\times$

Alternative fabrication techniques include direct solvent blending^35–38^, direct melt blending^35,39–41^, in-situ formation of matrix and particles^42–44^ and hybrid core-shell nanoparticles^45^. Our method is a hybrid approach of the two first methods, while additionally incorporating a freeze drying stage. The freeze drying process produces large amounts of micron-size, brittle granules (of single or multiple species), ready to be instantaneously fragmented in the polymer matrix at low-temperatures, in contrast to conventional blending. The efficiency in which nanoparticles are treated and dispersed, in combination with the established extrusion technique, constitute a direct and cost efficient method for large-scale production of nanocomposite films, with strong potential to tailor the optical properties of the end-product. We note that, in contrast to the work presented herein, none of the aforementioned fabrication techniques can lead to end-products featuring all the properties presented in Table 4.

## Conclusions

Strong potential for tailoring the optical properties of nanocomposite optical films has been exhibited via an elaborate bulk fabrication process. We have shown that, without compromising the uniformity of the nanocomposite, the method allows for tuning of the optical properties of the nanocomposite film by regulating the amount and type of active material embedded in the polymer matrix. In contrast to previous works, we have achieved uniform dispersion of preselected unary and heterogeneous combinations of nanoparticles in a polymeric matrix through the use of a freeze granulation process followed by a low temperature extrusion. Apart from offering insight in the fields of nanoparticle suspension and nanocomposite optical, thermal and mechanical properties, the produced films can be customized to fit a great number of industrial and everyday life applications. These include, but are not limited to, greenhouse covers, anti-glaring and/or anti-reflection coatings for glass surfaces, flat control displays and optical elements. Finally, the low fabrication cost is expected to be a strong competitive advantage.
